# Transcriptional and epigenetic reprogramming during androgenesis in *Brassica Rapa*

**DOI:** 10.1186/s43897-026-00250-x

**Published:** 2026-11-02

**Authors:** Hui Guo, Rongrong Xie, Jun Zhang, Di Wu, Mayra Ahmed, Yang Zhang, Hongbo Gao

**Affiliations:** Shanghai Collaborative Innovation Center of Agri-Seeds, Joint Center for Single Cell Biology, School of Agriculture and Biology, Shanghai Jiao Tong University, Shanghai, 200240 China

Androgenesis involves three key stages: cell reprogramming, totipotency acquisition, and embryogenesis initiation (Ahmadi et al. 2018). Under in vitro stress, late unicellular microspores can be diverted from gametophytic development toward embryogenesis, regenerating haploid or double-haploid plants (Perez-Perez et al. 2019). Despite its biotechnological value, microspore culture faces significant limitations, including low induction rates and failure to establish induction systems (Shumilina et al. 2015). Among these constraints, genotype is the most challenging factor, directly limiting induction efficiency even within the same species (Gao et al. 2024). Notably, only late unicellular microspores preceding pollen mitosis I are competent for heat-stress induction (Zaki and Dickinson 1990).

The regulatory network governing cellular reprogramming and embryogenesis remains poorly understood, owing to limited investigation into specific regulatory sites and the molecular mechanisms underlying germ cell fate transition (Corral-Martinez, Siemons et al. 2020). Several plant transcription factors have been identified as capable of reprogramming somatic cells into embryogenic, totipotent cells. For instance, BABY BOOM (BBM) from *Brassica napus*, when expressed specifically in microspores, can directly induce cell fate transition and trigger in vivo androgenesis, bypassing stress treatment (Boutilier et al. 2002). BBM, together with LEC1 and LEC2, can jointly induce somatic embryogenesis; the latter two, along with L1L, ABI3, and FUS3, constitute the LAFL network (Jia et al. 2014). Although ABI3 and FUS3 overexpression does not directly initiate somatic embryogenesis, it confers embryonic characteristics to seedlings (Gazzarrini et al. 2004). However, whether BBM and LAFL factors function within the same pathway remains unclear.

Epigenetic modifications—particularly DNA methylation and histone modifications—also play essential roles in regulating cellular differentiation and microspore development (Zhao et al. 2022). Microspore embryogenesis requires genome-wide DNA demethylation, histone hyperacetylation, and specific histone methylation modifications, collectively relaxing chromatin to enable transcription factor binding and gene activation (El-Tantawy et al. 2014). However, current research remains largely limited to genome-wide profiling, with little investigation into specific genomic loci and targeted gene regulation. Given that epigenetic modifications often orchestrate development by influencing a limited set of key target genes, fine-scale mapping of these modifications and identification of key regulatory factors are crucial for understanding cell fate transition mechanisms (Yun et al. 2012).Here, we investigated the transcriptional and epigenetic reprogramming during microspore embryogenesis in *Brassica rapa*, aiming to elucidate the molecular mechanisms underlying cell fate transition and induction efficiency. Our findings are expected to provide a foundation for enhancing haploid breeding efficiency and developing new germplasm.

To investigate androgenesis regulation and epigenetic dynamics during microspore embryogenesis, we performed RNA-seq on the inducible line K2 and non-inducible line 49CX across late unicellular microspores (lmsp), heat-treated microspores (hmsp), control microspores (mock-treated without heat stress, cmsp) , 2-cell, 4-cell, and 8-cell embryo stages, along with WGBS and H3K4me3 ChIP-seq across multiple developmental stages in both lines (Fig. 1A-B, Fig. S1). Principal component analysis (PCA) clearly segregated the transcriptomic profiles of microspores from those of leaf tissues. Strikingly, heat treatment triggered a substantial transcriptome reprogramming in the K2 microspores, positioning its transcriptomic profile closer to - and progressively refining it toward - early embryonic states. In contrast, control microspores (mock-treated without heat stress, cmsp) exhibited no transcriptomic divergence with heat-treated microspores(hmsp) in 49CX (Fig. 1C-D, Fig. S2). Differential expression analysis identified 3371 heat-responsive genes in K2 (1623 upregulated, 1748 downregulated), with downregulated genes enriched for pollen development and pollen tube growth functions, while upregulated genes included numerous developmental regulators. Notably, 1523 genes were uniquely heat-activated in K2, showing minimal overlap with control or 49CX samples (Fig. 1E). K-means clustering revealed distinct temporal patterns: cluster 1 (n=531) showed transient stress-responsive upregulation without sustained embryogenic expression, whereas clusters 2-5 (n=992) exhibited prolonged or progressively increasing expression throughout embryogenesis (Fig. 1F). GO analysis demonstrated that cluster 1 was enriched for stress response functions including HSP70-1 and HSP81-2, while clusters 2-5 were significantly enriched for biological regulation functions (n=215, p<0.005), containing key embryogenic regulators BBM, LEC1, and LEC2(Fig. S3). Network analysis of these 215 genes identified four interconnected modules activated upon heat treatment: phytohormone biosynthesis and signaling, stress response, embryogenesis regulators, and epigenetic remodeling factors, interconnected through hubs including BAK1, PYR1, and ABI3, which further activated downstream WRKY transcription factors (Fig. 1G).

**Fig. 1:**
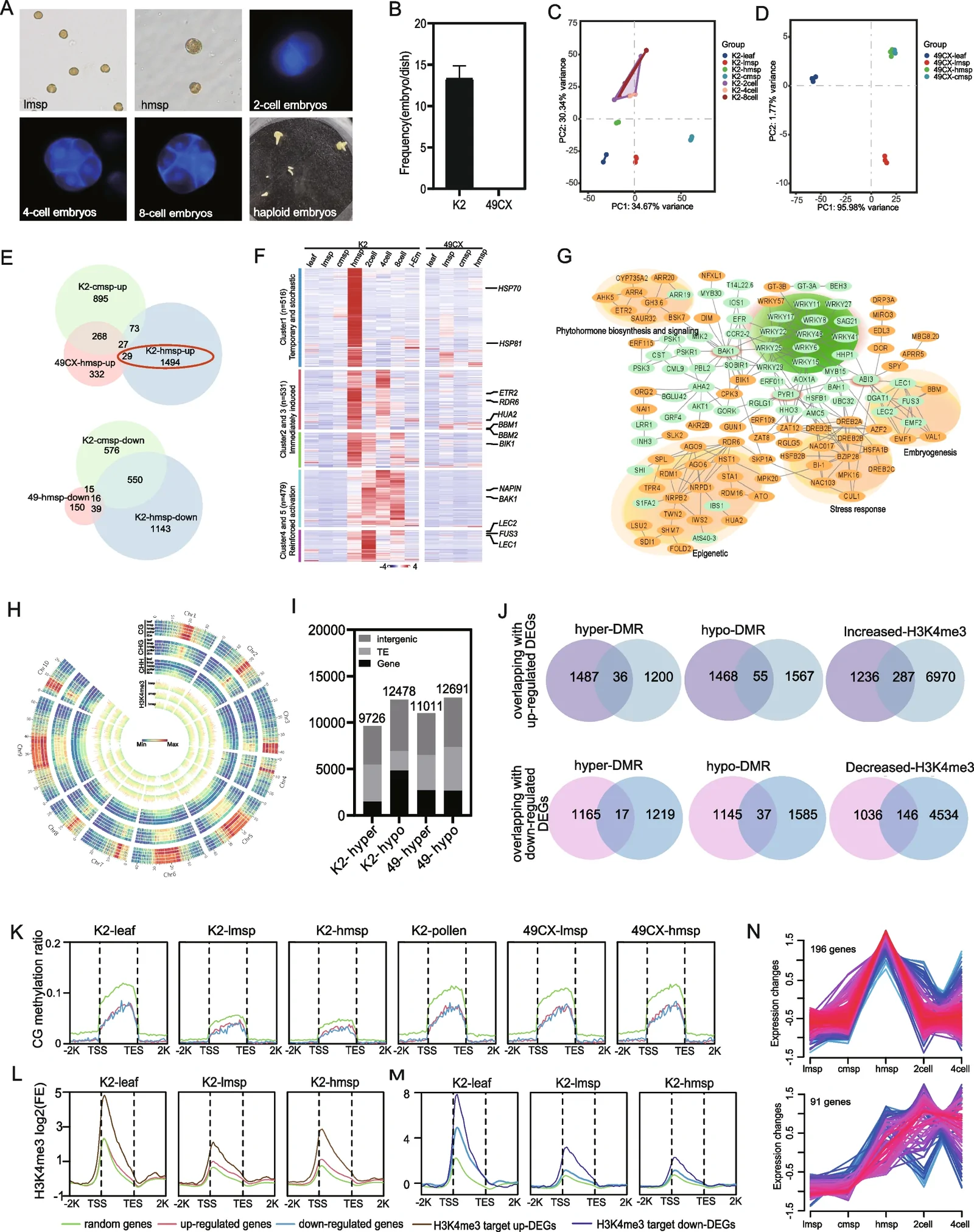
**A** The late unicellular microspores（lmsp）extracted from appropriately sized buds expanded after undergoing heat stress in vitro(hmsp), and subsequently divided into 2-cell embryos, 4-cell embryos, 8-cell embryosduring cultivation at 25°C, eventually developing into haploid embryos. **B** Bar chart showing the embryo production rates of different varieties in Brassica rapa (K2 and 49CX). Based on this, we define K2 as an inducible variety and 49CX as a non-inducible variety. **C-D** PCA plot showing the clustering between three replicates of different samples for RNA-seq analysis in K2(**C**) and 49CX(**D)**.Under heat stress, the gene expression profiles of K2-hmsp and K2-cmsp diverged markedly, whereas those of 49CX-hmsp and 49CX-cmsp remained largely similar. **E** Venn diagram analysis of differentially expressed genes (DEGs) in heat-stressed microspores (K2-hmsp, 49CX-hmsp) or mock-treated K2 microspores (K2-cmsp) versus untreated late-stage uni-cellular microspores (K2-lmsp or 49CX-lmsp) with the criteria of |log2FC| > 2, padj < 0.05， the red circle indicates 1523 uniquely heat-activated genes in K2 heat-treated microspores (hmsp). **F** Hierarchical clustering of 1523 heat-induced genes in K2 heat-stressed microspores(hmsp) across various stages of induction. Heatmap highlights distinct expression patterns between K2 and 49CX. **G** Functional association network of 215 heat-induced genes involved in biological regulation. Genes are labeled with their Arabidopsis ortholog names, and key pathways—including phytohormone biosynthesis and signaling, stress response, embryogenesis, and epigenetic remodeling—are highlighted. Orange denotes genes from the immediate response category (clusters 2 and 3), while green marks those with reinforced activation (clusters 4 and 5). **H** Heatmaps of genome-wide DNA methylation and H3K4me3 enrichment across five K2 tissues (lmsp, cmsp, hmsp, i-Em, leaf), calculated in 10-kb windows and normalized per context. **I** Bar chart displaying the distribution of hyper- and hypo-DMRs in K2 and 49CX before and after heat treatment. The proportions of DMRs overlapping gene, TE (transposable element), and intergenic regions were shown. **J** Venn diagrams showing the overlap of heat-induced upregulated genes (1,523) with hyper-DMRs, hypo-DMRs, or increased H3K4me3 peaks, and downregulated genes (1,182) with hyper-DMRs, hypo-DMRs, or decreased H3K4me3 peaks. **K** Gene body methylation profiles of the 1523 specifically up-regulated genes, 1182 specifically down-regulated genes and control genes (n=2000) in leaf, late-stage unicellular microspores(lmsp), heat-induced microspores (hmsp) and pollen in K2 or 49CX lines. **L** H3K4me3 enrichment around all specifically up-regulated genes (n=1523) or those overlapping with increased H3K4me3 peaks (n=287) and control genes (n=2000) in leaf, lmsp, hmp of K2. **M** H3K4me3 enrichment around all specifically down-regulated genes (n=1182) or those overlapping with decreased H3K4me3 peaks (n=146) and control genes (n=2000) in leaf, lmsp, hmp of K2. **N** Gene expression trend graph showing two different expression patterns of 287 up-regulated genes with increased H3K4me3 peaks during microspore induction

Global methylation patterns were consistent, showing high levels in centromeric regions and low levels in chromosomal arms. Notably, WGBS analysis revealed that heat induction triggered a pronounced CG methylation reduction specifically in inducible K2, particularly over centromeric and transposable element (TE) regions, whereas no such reduction occurred in non-inducible 49CX microspores. CHG and CHH methylation remained low and unchanged (Fig. 1H, Fig. S4C). Differentially methylated region (DMR) analysis between K2 hmsp and lmsp identified 9726 hyper-DMRs and 12478 hypo-DMRs, with hypo-DMRs preferentially localized to gene-associated regions (Fig. 1I). Interestingly, despite the lack of global methylation changes in 49CX, a comparable number of DMRs in the gene body region were detected, with 10.8% of hyper-DMRs and 12.9% of hypo-DMRs overlapping those in K2 (FigS4A-B). Correlation analysis showed only minimal overlap between DMRs and differentially expressed genes (DEGs) within 200 bp flanking regions: merely 36 (2.4%) of upregulated genes co-localized with hyper-DMRs and 55 (3.6%) with hypo-DMRs (Fig. 1J). However, global methylation analysis revealed a significant reduction in gene body methylation in inducible microspores, with heat stress causing further depletion. This pattern was absent in non-inducible microspores. Notably, both up- and down-regulated genes exhibited below-average gene body methylation levels (Fig. 1K). Additionally, we found that K2 leaf tissues exhibited lower expression of RNA-directed DNA methylation (RdDM) pathway genes than 49CX, though microspore expression profiles showed no significant varietal differences despite individual gene fluctuations (Fig. S4D). We propose that inherently low gene-body methylation confers transcriptional plasticity, enabling flexible expression regulation under stress.

The global H3K4me3 level showed minimal changes in heat-treated microspores (hmsp) compared to untreated controls (cmsp) in K2 but significant redistribution, identifying 12137 differential peak regions—most within 1 kb upstream of transcription start sites (Fig. S5A-B). Among these, 384 peaks were significantly upregulated (log2(fold change) >2, P < 0.05) and 124 were downregulated, a marked difference from control treatments that showed only 37 upregulated and 36 downregulated peaks. H3K4me3-marked genes included multiple transcription factors involved in embryonic development and cell fate determination, such as WRKY25 and WRKY61 (Fig. S5C). We next examined the correlation between H3K4me3 variation and gene expression during microspore culture. Among the 1523 upregulated genes, 287 (18.8%) overlapped with heat-induced H3K4me3 targets, while 146 (12.4%) of 1182 downregulated genes overlapped with heat-decreased H3K4me3 targets (Fig. 1J). GO enrichment analysis revealed that these 287 genes were enriched for regulation of biological process" and "metabolic processes" (Fig. S7). Additionally, they showed higher basal H3K4me3 levels than other DEGs or random controls, and H3K4me3 changes correlated with expression direction (Fig. 1L-M). Expression trend graph showed the 287 targeted upregulated genes revealed two patterns: 196 genes showed rapid heat-responsive activation followed by quick attenuation, while 91 exhibited progressive upregulation peaking at 2-cell or 4-cell embryo stages (Fig. 1M). Although classic regulators like BBM and LEC1 lacked heat-induced H3K4me3 changes, other rapidly responsive regulators including bHLH49, NAC087, WRKY25, and WRKY61 displayed strong H3K4me3-transcription correlations (Fig. S6). Thus, H3K4me3 specifically targets and regulates developmental gene networks involved in transcriptional reprogramming during microspore induction, providing both immediate activation signals for rapid-response genes and sustained expression maintenance into later embryonic stages.

Collectively, these results demonstrate that heat induction triggers coordinated epigenetic reprogramming involving DNA methylation changes and H3K4me3 redistribution, low methylation allows embryo-related genes to flexibly adjust expression, while H3K4me3 marks key transcription factors and developmental genes, thereby driving the microspore cell fate transition from gametophyte development to embryogenesis.

The transcriptional and epigenetic mechanisms underlying cell fate transition during microspore embryogenesis remain to be elucidated. At the molecular level, our RNA-seq analysis of *Brassica* species revealed rapid transcriptome activation following heat stress, including the synchronized initiation of key embryogenic regulators (BBM, LEC1, LEC2), hormone signaling pathways, and epigenetic remodeling machinery, providing a more precise and dynamic expression profile. A critical unresolved question is whether such transcriptional activation represents a universal feature of late-stage microspores or is restricted to specific subpopulations, a distinction requiring single-cell sequencing technologies. Furthermore, the mechanistic roles of embryogenic regulators remain debated, necessitating cross-species comparative studies to elucidate the conservation and divergence of plant embryogenic regulatory networks, ultimately enabling the modulation of transcriptional accessibility to enhance induction efficiency. At the epigenetic level, consistent with studies in *Brassica* and barley, we observed that heat stress triggers genome-wide hypomethylation, with variably expressed genes exhibiting significantly lower gene-body methylation, supporting a model in which transcriptional plasticity and locus-specific epigenetic modifications jointly regulate cell fate transition. Histone modifications also profoundly influence embryogenesis efficiency (Li et al. 2014, Rymen et al. 2019), and our data further establish H3K4me3 as an active regulator during androgenesis. However, the interplay between H3K4me3 and other histone modifications, particularly H3K27me3, and their integration with additional epigenetic regulatory layers remain to be fully resolved. Future studies should focus on how these regulatory layers coordinately drive transcriptional reprogramming during microspore embryogenesis.

## Supplementary Information

Supplementary Material 1.

Supplementary Material 2.

Supplementary Material 3.

## Data Availability

The obtained sequencing data that support the findings of this study have been deposited in the National Center for Biotechnology Information Gene Expression Omnibus (GEO) database.
