# Supplementary material for: Transcriptional and epigenetic reprogramming during androgenesis in Brassica Rapa
Source: Mol Hortic. 2026 Nov 2;6:80. doi: 10.1186/s43897-026-00250-x (PMC13726228; doi:10.1186/s43897-026-00250-x)
Supplement: Supplementary file 1 [file 43897_2026_250_MOESM2_ESM.pdf]

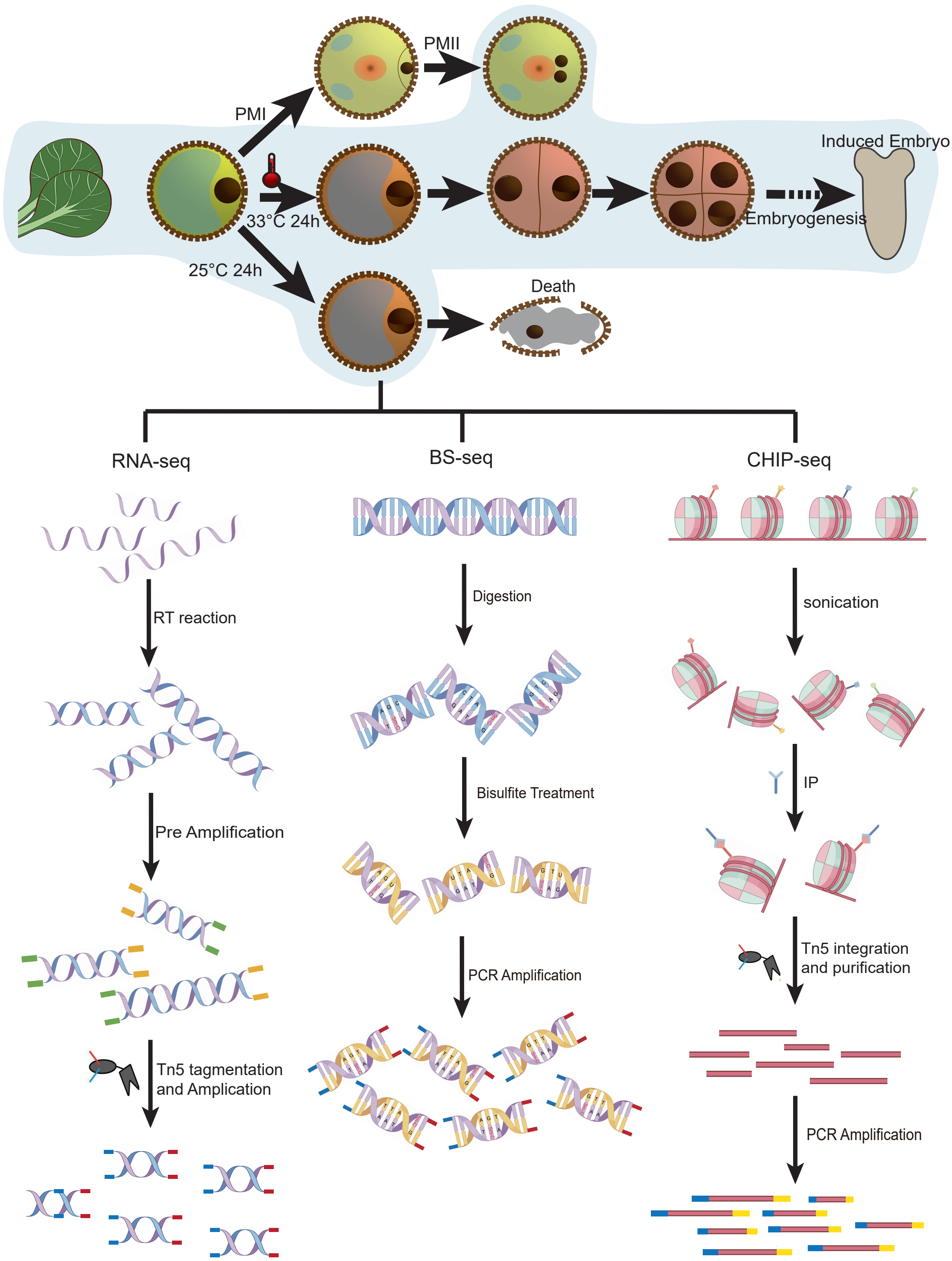

FigS1.Schematic workflow of multi-omics analysis during microspore embryogenesis induction

This figure systematically illustrates the key experimental procedures and technical workflow for integrating transcriptomics (RNA-seq), DNA methylomics (BS-seq), and chromatin immunoprecipitation sequencing (ChIP-seq) during the induction of microspore embryogenesis. First, microspores are subjected to temperature treatments at either 33°C or 25°C for 24 hours to induce embryogenic differentiation or programmed cell death (Dead), respectively.For transcriptome analysis, collected RNA samples undergo reverse transcription (RT), pre-amplification, and Tn5-mediated tagmentation to construct high-throughput sequencing libraries. DNA methylation analysis is based on bisulfite sequencing (BS-seq), which includes enzymatic digestion of genomic DNA, bisulfite conversion, and subsequent PCR amplification.

The chromatin immunoprecipitation sequencing (ChIP-seq) workflow involves chromatin shearing via sonication, specific antibody-based immunoprecipitation (IP), Tn5 transposase-mediated fragmentation and adapter integration, and final library amplification and purification.This integrated analytical strategy captures multi-dimensional information, including transcriptional dynamics, DNA methylation modifications, and histone modifications, providing comprehensive methodological support for systematically elucidating the epigenetic reprogramming and transcriptional regulatory mechanisms during cell fate transition in microspores.

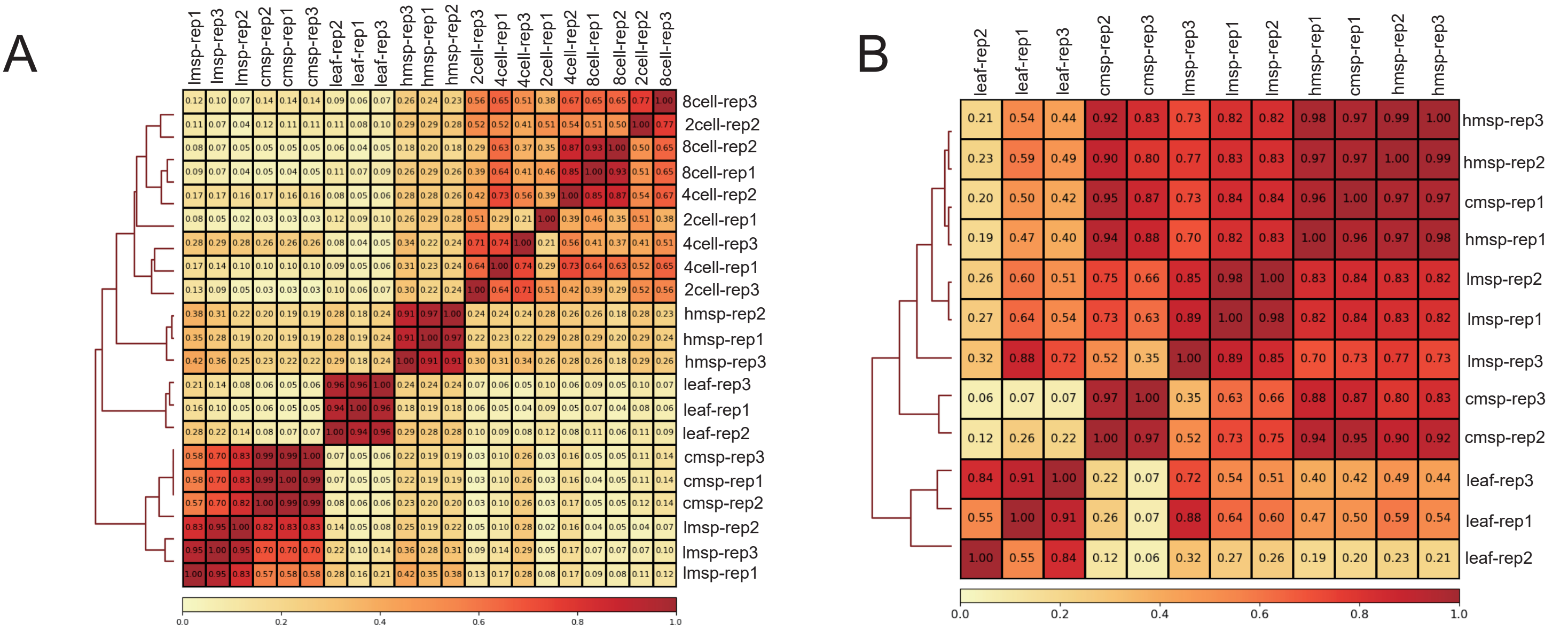

FigS2.The sample correlations for RNA-seq analysis

Heatmap showing the sample correlation for RNA-seq analysis in K2(**A**) and 49CX(**B**). Scale bar represents the correlation coefficient.The transcriptomic data of K2-lmsp,K2-hmsp and K2-cmsp show clear classification, with distinct expression patterns and strong correlations among the three biological replicates within each treatment. This indicates high reproducibility and stability in its gene expression responses across different treatments.In contrast, the gene expression profiles of 49CX-lmsp,49CX-hmsp and 49CX-cmsp exhibit high similarity, this suggests that this line has a blunted transcriptional response to different induction treatments and fails to display the distinct treatment-specific expression changes observed in K2.

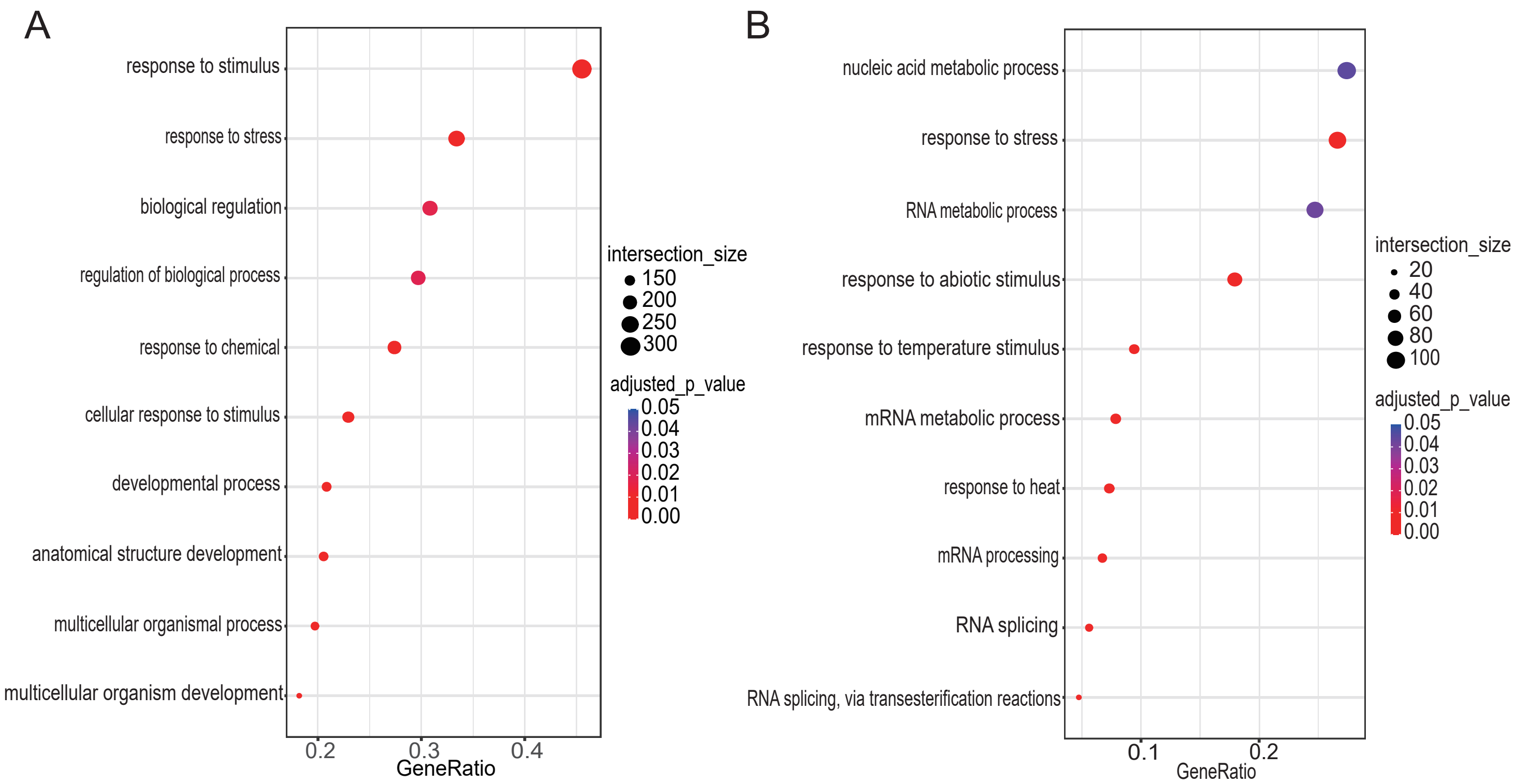

FigS3.Gene Ontology analysis of 1523 heat-activated genes in K2

Bubble diagram showing the Gene Ontology analysis of cluster2, cluster3, cluster4, cluster5(**A**) and cluster1(**B**).GO enrichment analysis indicates that the genes in Cluster 1 and Cluster 2-5 exhibit significant functional differentiation and ordered coordination. The genes in Cluster 1 are notably enriched in pathways related to environmental signal perception, transcriptional regulation, and RNA metabolism, suggesting their primary role in early stress response and transcriptional/post-transcriptional regulation. They provide a rapid and dynamic molecular foundation for cell fate transition.In contrast, the genes in Cluster 2-5 are enriched in functions associated with developmental regulation and multicellular tissue construction, indicating that this gene group is more inclined to participate in biological processes such as cell fate determination, tissue morphogenesis, and coordinated multicellular development. These genes likely drive structural formation and organ differentiation during the middle to late stages of embryogenesis.Based on these findings, it is hypothesized that in K2-hmsp, the genes in Cluster 1 may rapidly initiate heat shock response and epigenetic reprogramming, thereby activating the developmental programs governed by Cluster 2-5.

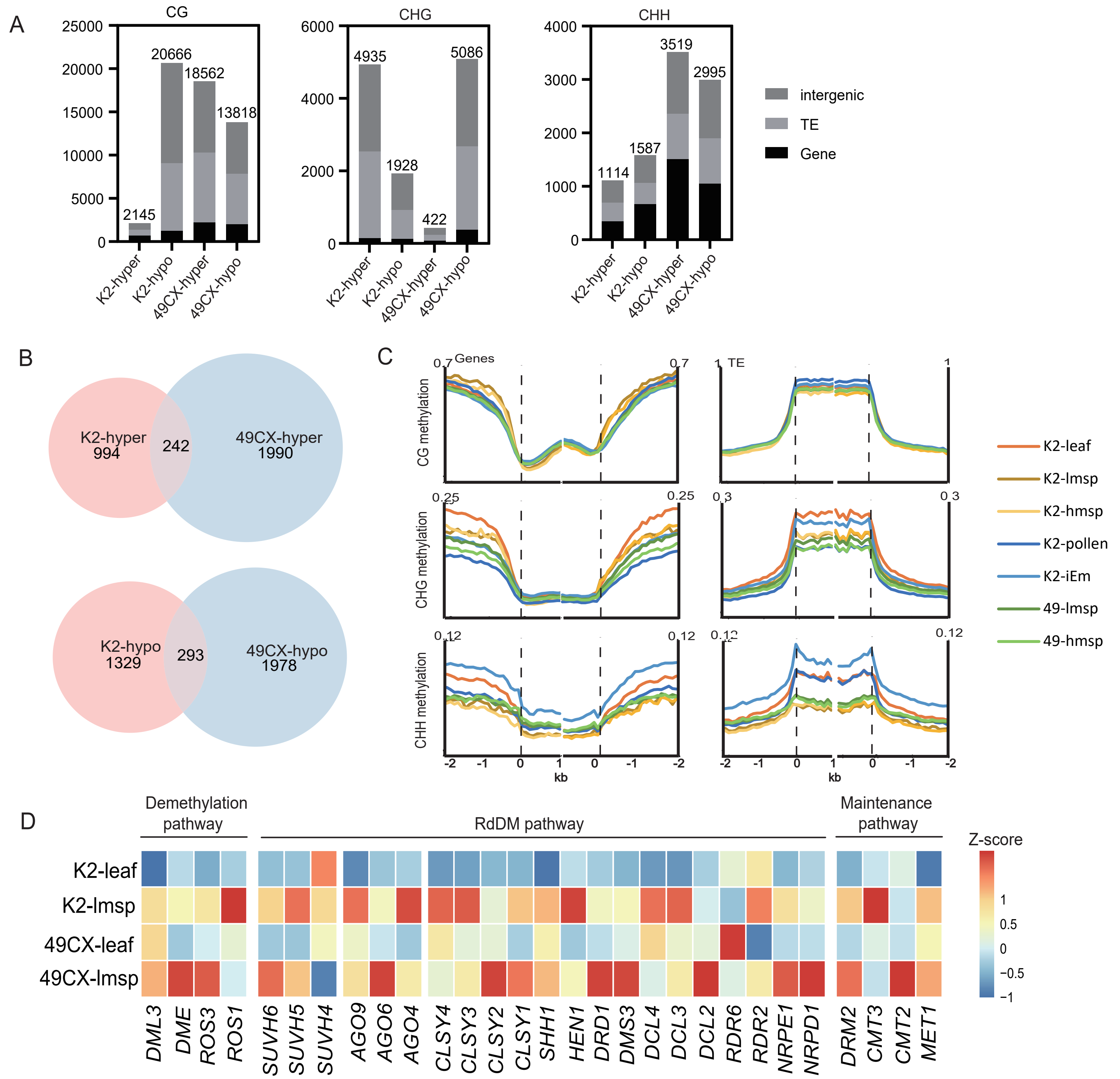

FigS4.Genotype-specific DNA methylation dynamics in microspore induction

A, Bar chart displaying the distribution of hyper- and hypo-DMRs(CG, CHG, and CHH) in K2 and 49CX before and after heat treatment. The proportions of DMRs overlapping gene, TE (transposable element), and intergenic regions were shown.

B,Venn diagram analysis of hyper-(A) and hypo-(B) differentially methylated region (DMR) in the gene body region of heat-stressed microspores(K2-hmsp, 49CX-hmsp) with the criteria of |meth.diff| > 10, padj < 0.01. There was 10.8% of hypermethylated DMRs and 12.9% of hypomethylated DMRs in 49CX-hmsp overlap with those in the K2 line.

C.Genes (left) and transposons (right) were aligned at the 5' end (left subpanels) or the 3' end (right subpanels) with average methylation in the CG, CHG or CHH context for each 100-bp interval were plotted for leaf, lmsp, hmsp, pollen, iEm of K2 and 49CX.In K2-hmsp, the methylation level at CG sites decreased significantly, particularly in centromeric and transposon (TE) regions. In contrast, no similar reduction in methylation levels was detected in microspores of the non-inducible variety 49CX. Moreover, in both K2 and 49CX, the methylation levels in CHG and CHH contexts remained low in both hmsp and lmsp regions, with no significant changes observed.

D,Heatmap showing transcriptome levels of key component genes of the maintenance pathways, RdDM pathway and demethylation pathway in leaf, lmsp of K2 and 49CX. Scale bar represents the z-score of the normalized expression values.

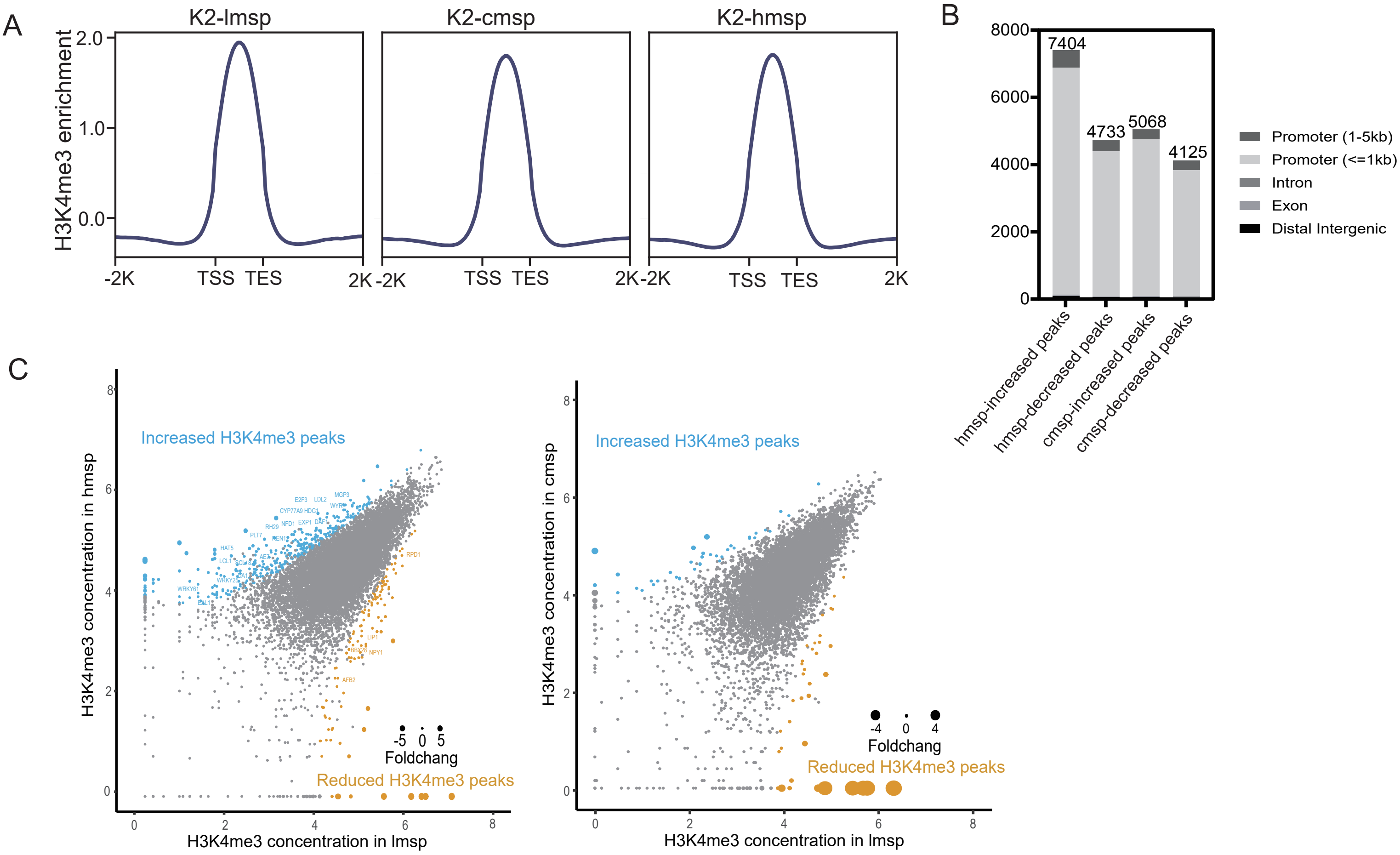

FigS5.Heat-Induced H3K4me3 remodeling at gene regions in inducible microspores

A, Fold-enrichment of all H3K4me3 peaks of all rigions in lmsp, cmsp and hmsp of the inducible line (K2).

B, Bar chart comparing the number of increased and decreased H3K4me3 peaks following heat treatment (hmsp) versus mock treatment(cmsp), showing their genomic distribution relative to gene regions.

C, Scatterplot illustrating the dynamic fluctuation of H3K4me3 peak in hmsp compared with lmsp (left) and in cmsp compared with lmsp (right) in K2 line, with the significant (padj<0.05) increased or reduced peak highlighted in blue and orange, respectively. Several genes that are targeted by these variable H3K4me3 peak were labeled.Among them are multiple transcription factors involved in embryonic development and cell fate determination.

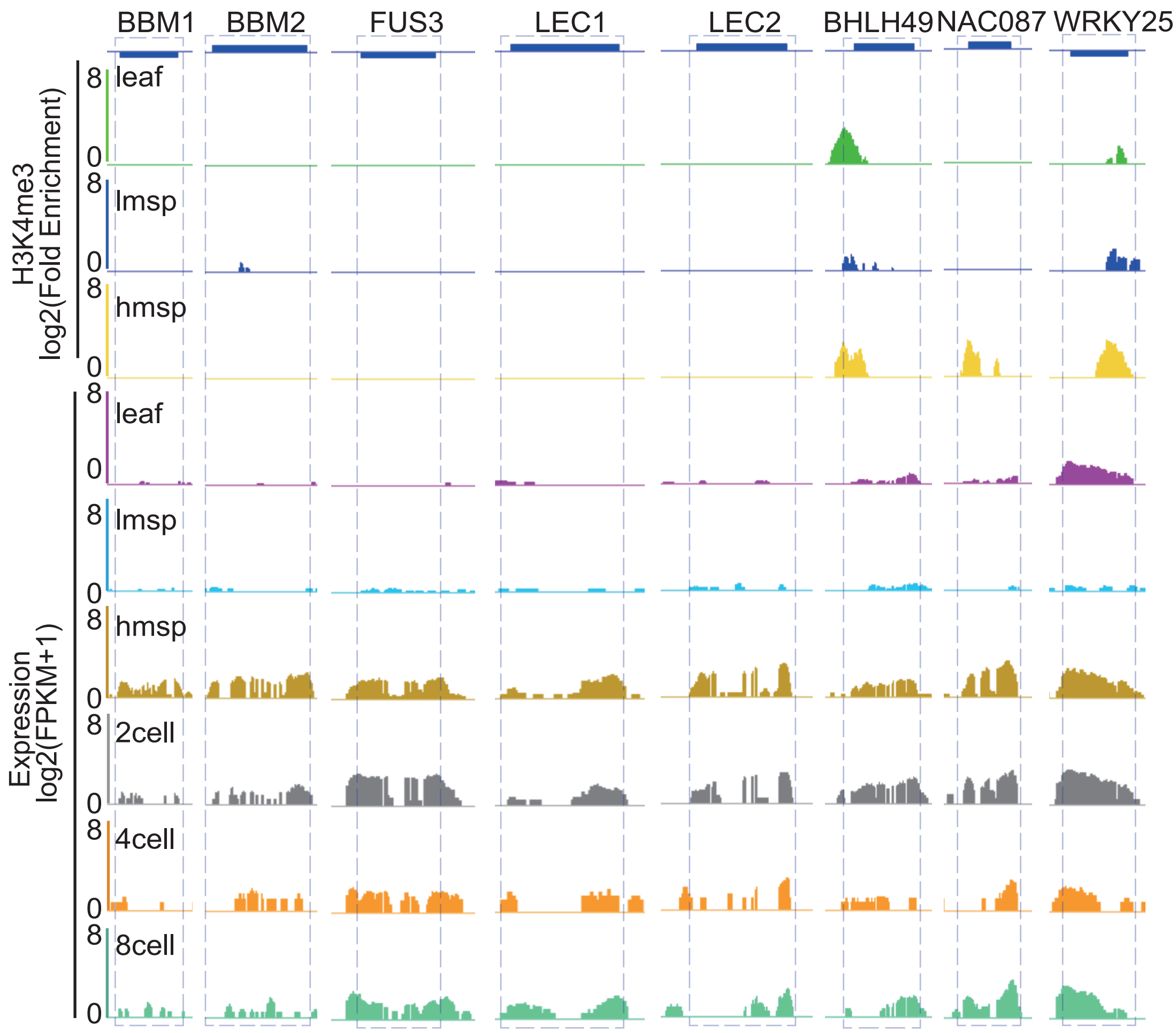

FigS6.Genomic snapshot of expression and H3K4me3 profile

Genomic snapshot of expression and H3K4me3 profile of several androgenesis-induced genes in multiple cell types including leaf, late-stage unicellular microspores(lmsp), heat-induced microspores (hmsp) and induced embryo structure at 2-, 4- or 8-cell stage of K2 line.In K2-hmsp, classical embryonic development regulators such as BBM and LEC1 showed no changes in H3K4me3 levels. However, significant correlations were observed between the upregulation of developmental regulators including bHLH49, NAC087, and WRKY25 and H3K4me3 deposition.

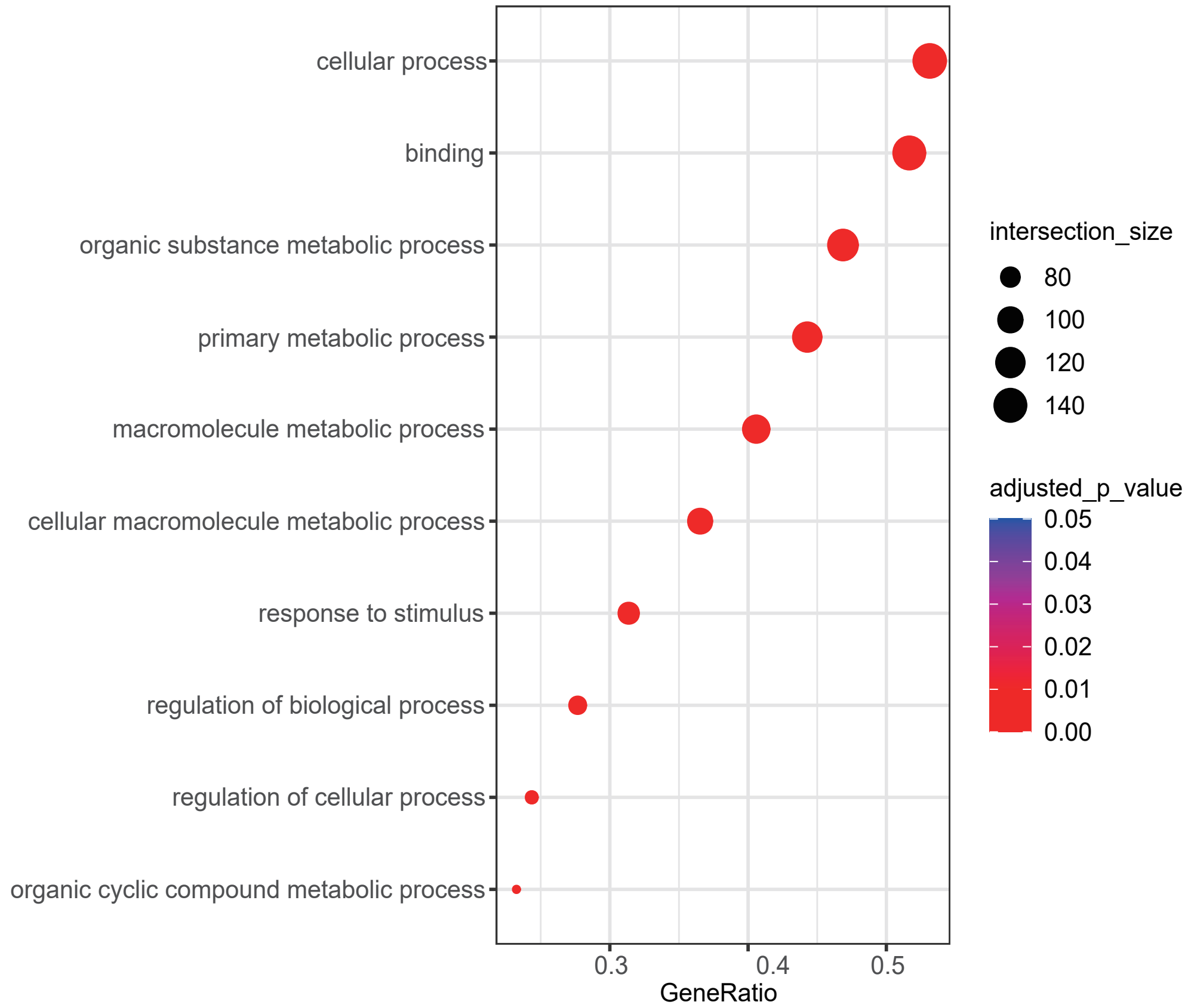

FigS7.GO enrichment analysis of 287 H3K4me3-modified upregulated genes
These 287 genes harboring H3K4me3 modifications showed significant enrichment in GO functional categories such as "regulation of biological process" and "metabolic process." This enrichment pattern indicates that these genes may not only be broadly involved in regulating fundamental cellular activities but could also influence reproductive developmental processes by modulating metabolic pathways.
