# Supplementary material for: Transcriptional and epigenetic reprogramming during androgenesis in Brassica Rapa
Source: Mol Hortic. 2026 Nov 2;6:80. doi: 10.1186/s43897-026-00250-x (PMC13726228; doi:10.1186/s43897-026-00250-x)
Supplement: Supplementary file 2 [file 43897_2026_250_MOESM1_ESM.docx]

**Materials and method**

**Plant materials and growth conditions**

The seeds of “49CX” and “K2” of Non-heading Chinese cabbage（*Brassica rapa.* ssp. *chinensis*）were sown in Petri dishes that were filled with water-soaked filter paper for germination at 20 °C for 2 days. The germinated seeds of plants were planted in pots containing nutrition soil with matrix and vermiculite (3:1:1 ratio) directly. The plants were grown under 16-h light/8-h dark in a growth chamber at 20 °C, with 75% relative humidity. Four weeks later, four-leaf stage seedlings were moved to another growth room for cold treatment at 4 °C (16 h light/8 h dark, 75% humidity) for two weeks, then move to the greenhouse at 20 °C under 16-h light/8-h dark.

**Microspore culture**

The microspore culture procedure used here was a modified version of that described before (Hoseini, Ghadimzadeh et al. 2014, Shumilina, Shmykova et al. 2015, Zhang, Zhang et al. 2016). Floral buds with a length of 2.5mm to 3mm containing a mixture of mid-to late unicellular microspore were harvested from the main and the first lateral branches of donor plants. These buds were surface sterilized by sodium hypochlorite (NaClO) 3.5% (w/v) solution for 15min with gentle shaking. The sterilized buds were then rinsed three times by sterilized distilled water (5 min each time). The surface sterilized buds were crushed in 5 mL of NLN medium(Lichter 1982) containing 13% (w/v) sucrose (pH 5.8) using a sterile glass rod. The crude suspension was filtered through 40 mm cell sieve, collected into 50 mL centrifuge tubes. Then, the volume was adjusted to 20 mL with NLN medium in 50 mL centrifuge tubes and the tubes were centrifuged at 100g for 10 min. The pellet was rinsed in fresh NLN medium. This procedure was repeated three times. The pellet can also be frozen into the -80°C freezer for subsequent library construction. The microspore density was adjusted to 1 × 105 microspores/mL using a hemocytometer. Then the microspore suspension was divided into 60 mm × 15 mm sterile glass Petri dishes, 5 mL per dish. The cultures were incubated at 33°C or 25°C for 1 d. Microspores incubated for 1 d at 33°C(heat-treated microspores) and 25°C(cold-treated microspores) were transferred to 15 ml centrifuge tubes, centrifuged at 100 g for 10 minutes, removed the supernatant, and the pellet was frozen in a -80°C freezer. Heat-treated microspores transferred to 25°C in the dark. Once the embryos became visible to the naked eye (approximately 400–500 mm long, about 12 d following microspore culture), the culture dishes were transferred to a rotary shaker in the dark at 50 rpm, 25°C.

**RNAseq-library construction and transcriptome Analysis**

Strand-specific RNA-sequencing libraries were prepared according to the protocol described before(Picelli, Faridani et al. 2014), from three biological replicates of lmsp, hmsp, cmsp, leaves of K2 and 49CX, respectively. The libraries were sequenced for paired-end reads of 150 bp using the Illumina Nova seq 6000 platform, sequencing depth 50◊. Trim the raw reads using Trim-Galore(Ooi, Mantalas et al. 2017) with the setting ‘-p 20’ followed by mapping to the reference genome of NHCC001 using hisat2(Pertea, Kim et al. 2016) with the setting ‘--paired --quality 20 --gzip’, and remove the PCR duplicated fragments using Picard(Tsyganov, James Perry et al. 2018). Perform the raw count processing and subsequent analysis of the differentially expressed genes (DEG) with DESeq2(Love, Huber et al. 2014) with the setting ‘padj < 0.05 & log2FoldChange) >2’. Perform clustering of the gene expression with the Pheatmap package, and visualize in an expression heatmap. The RPKM (reads per kilobase per million mapped reads) values of the genes and the TEs were calculated with Stringtie(Pertea, Kim et al. 2016).

**Histone modification analysis by CHIP-SEQ**

CHIP-SEQ libraries were generated as described previously(Villar and Köhler 2010), and sequenced on Illumina Nova seq 6000 platform, sequencing depth 50◊ (Genewiz, China)(Nakato and Sakata 2021). Paired-end data was removed the fragment joints by Trim-Galore(Ooi, Mantalas et al. 2017) and then alignment to genome NHCC001(Li, Liu et al. 2020) by Bowtie2(Langdon 2015). Use Samtools(Li, Handsaker et al. 2009) to convert the sam file from the previous step into a bam file, sort the bam file, and filter out the single read, mitochondrial read, and chloroplast read. Merge bam files from two replications. Peaks were then called using MACS2(Zhang, Liu et al. 2008), with the setting ‘-f BAMPE -B -q 0.01 --keep-dup=all’. Chipseeker(Yu, Wang et al. 2015, Wang, Li et al. 2022) used for annotate the results of peak calling. Diffbind(Ross-Innes, Stark et al. 2012) is used for peak enrichment comparison, including overlapping and merging peak sets, counting sequencing reads overlapping intervals in peak sets, and identifying statistically significantly differentially bound sites based on evidence of binding affinity.

**Bisulfite Sequencing Library Construction and Whole-Genome Bisulfite Sequencing**

Cell lysis and bisulfite conversion were performed using the ZYMO EZ DNA Methylation-Gold kit (ZYMO RESEARCH, USA) according to the manufacturer's instructions, bisulfite conversion efficiency > 99%. Paired-end (150 bp) bisulfite-sequencing libraries for Illumina sequencing were constructed as described earlier(Smallwood, Lee et al. 2014). Adaptor-ligated DNA was enriched by 13 cycles of PCR with the following thermal profile: an initial incubation at 95 ◦C for 2 min, 13 cycles at 95 ◦C for 30 s, 65 ◦C for 20 s, 72 ◦C for 30 s and a final incubation at 72 ◦C for 3 min. The enriched libraries were purified with the Ampure XP beads (BECKMAN) prior to quantification with Bioanalyzer (Agilent 5200). Bisulfite libraries were constructed from two biological replicates of each sample. Sequence was performed at the Illumina Nova seq 6000 platform of GENEWIZ biotechnology Co., Ltd, sequencing depth 50◊. The low-quality sequencing reads, adaptor sequences and the first 9 bp of each read were trimmed using Trim-Galore(Ooi, Mantalas et al. 2017). Trimmed and high-quality reads were then mapped to the reference genome of NHCC001(Li, Liu et al. 2020) using BSmap 1.0 (https://github.com/genome-vendor/bsmap)(Xi and Li 2009). Methylated cytosines were reported and only cytosines with more than 5 informative sequencing reads were retained for further study. The methylkit package is used to identify differentially methylated regions (DMRs) with the setting ‘qvalue < 0.01 & meth.diff > 10’ and annotate them.

**References**

Hoseini, M., M. Ghadimzadeh, B. Ahmadi and J. A. Teixeira da Silva (2014). "Effects of ascorbic acid, alpha-tocopherol, and glutathione on microspore embryogenesis in Brassica napus L." In Vitro Cellular & Developmental Biology - Plant **50**(1): 26-35.

Langdon, W. B. (2015). "Performance of genetic programming optimised Bowtie2 on genome comparison and analytic testing (GCAT) benchmarks." BioData Min **8**(1): 1.

Li, H., B. Handsaker, A. Wysoker, T. Fennell, J. Ruan, N. Homer, G. Marth, G. Abecasis and R. Durbin (2009). "The Sequence Alignment/Map format and SAMtools." Bioinformatics **25**(16): 2078-2079.

Li, Y., G.-F. Liu, L.-M. Ma, T.-K. Liu, C.-W. Zhang, D. Xiao, H.-K. Zheng, F. Chen and X.-L. Hou (2020). "A chromosome-level reference genome of non-heading Chinese cabbage [Brassica campestris (syn. Brassica rapa) ssp. chinensis]." Horticulture Research **7**(1): 212.

Lichter, R. (1982). "Induction of Haploid Plants From Isolated Pollen of Brassica napus." Zeitschrift für Pflanzenphysiologie **105**(5): 427-434.

Love, M. I., W. Huber and S. Anders (2014). "Moderated estimation of fold change and dispersion for RNA-seq data with DESeq2." Genome Biol **15**(12): 550.

Nakato, R. and T. Sakata (2021). "Methods for ChIP-seq analysis: A practical workflow and advanced applications." Methods **187**: 44-53.

Ooi, C. C., G. L. Mantalas, W. Koh, N. F. Neff, T. Fuchigami, D. J. Wong, R. J. Wilson, S. M. Park, S. S. Gambhir, S. R. Quake and S. X. Wang (2017). "High-throughput full-length single-cell mRNA-seq of rare cells." PLoS One **12**(11): e0188510.

Pertea, M., D. Kim, G. M. Pertea, J. T. Leek and S. L. Salzberg (2016). "Transcript-level expression analysis of RNA-seq experiments with HISAT, StringTie and Ballgown." Nat Protoc **11**(9): 1650-1667.

Picelli, S., O. R. Faridani, A. K. Björklund, G. Winberg, S. Sagasser and R. Sandberg (2014). "Full-length RNA-seq from single cells using Smart-seq2." Nat Protoc **9**(1): 171-181.

Ross-Innes, C. S., R. Stark, A. E. Teschendorff, K. A. Holmes, H. R. Ali, M. J. Dunning, G. D. Brown, O. Gojis, I. O. Ellis, A. R. Green, S. Ali, S.-F. Chin, C. Palmieri, C. Caldas and J. S. Carroll (2012). "Differential oestrogen receptor binding is associated with clinical outcome in breast cancer." Nature **481**(7381): 389-393.

Shumilina, D. V., N. A. Shmykova, L. L. Bondareva and T. P. Suprunova (2015). "Effect of genotype and medium culture content on microspore-derived embryo formation in Chinese cabbage (Brassica rapa ssp. chinensis) cv. Lastochka." Biology Bulletin **42**(4): 302-309.

Smallwood, S. A., H. J. Lee, C. Angermueller, F. Krueger, H. Saadeh, J. Peat, S. R. Andrews, O. Stegle, W. Reik and G. Kelsey (2014). "Single-cell genome-wide bisulfite sequencing for assessing epigenetic heterogeneity." Nature Methods **11**(8): 817-820.

Tsyganov, K., A. James Perry, S. Kenneth Archer and D. Powell (2018). "RNAsik: A Pipeline for complete and reproducible RNA-seq analysis that runs anywhere with speed and ease." The Journal of Open Source Software **3**: 583.

Villar, C. B. and C. Köhler (2010). "Plant chromatin immunoprecipitation." Methods Mol Biol **655**: 401-411.

Wang, Q., M. Li, T. Wu, L. Zhan, L. Li, M. Chen, W. Xie, Z. Xie, E. Hu, S. Xu and G. Yu (2022). "Exploring Epigenomic Datasets by ChIPseeker." Current Protocols **2**(10).

Xi, Y. and W. Li (2009). "BSMAP: whole genome bisulfite sequence MAPping program." BMC Bioinformatics **10**(1): 232.

Yu, G., L.-G. Wang and Q.-Y. He (2015). "ChIPseeker: an R/Bioconductor package for ChIP peak annotation, comparison and visualization." Bioinformatics **31**(14): 2382-2383.

Zhang, L., Y. Zhang, Y. Gao, X. Jiang, M. Zhang, H. Wu, Z. Liu and H. Feng (2016). "Effects of histone deacetylase inhibitors on microspore embryogenesis and plant regeneration in Pakchoi ( Brassica rapa ssp. chinensis L.)." Scientia Horticulturae **209**: 61-66.

Zhang, Y., T. Liu, C. A. Meyer, J. Eeckhoute, D. S. Johnson, B. E. Bernstein, C. Nusbaum, R. M. Myers, M. Brown, W. Li and X. S. Liu (2008). "Model-based Analysis of ChIP-Seq (MACS)." Genome Biology **9**(9): R137.
